# Experiences of care in the context of payment for performance (P4P) in Tanzania

**DOI:** 10.1186/s12992-019-0503-9

**Published:** 2019-10-16

**Authors:** Victor Chimhutu, Marit Tjomsland, Mwifadhi Mrisho

**Affiliations:** 1 Department of Health Promotion and Development, University of Bergen, P.O Box 7807, 5020 Bergen, Norway; 2 Department of Social Science, Faculty of Education, Western Norway University of Applied Sciences, P.O Box 7030, 5020 Bergen, Norway; 3 Ifakara Health Institute, P.O Box 78373, Dar es Salaam, Tanzania

**Keywords:** Pay for performance (P4P), Results-based financing, Performance-based financing, Output-based financing, Tanzania, Africa

## Abstract

**Background:**

Tanzania is one of many low income countries committed to universal health coverage and Sustainable Development Goals. Despite these bold goals, there is growing concern that the country could be off-track in meeting these goals. This prompted the Government of Tanzania to look for ways to improve health outcomes in these goals and this led to the introduction of Payment for Performance (P4P) in the health sector. Since the inception of P4P in Tanzania a number of impact, cost-effective and process evaluations have been published with less attention being paid to the experiences of care in this context of P4P, which we argue is important for policy agenda setting. This study therefore explores these experiences from the perspectives of health workers, service users and community health governing committee members.

**Methods:**

A qualitative study design was used to elicit experiences of health workers, health service users and health governing committee members in Rufiji district of the Pwani region in Tanzania. The Payment for Performance pilot was introduced in Pwani region in 2011 and data presented in this article is based on this pilot. A total of 31 in-depth interviews with health workers and 9 focus group discussions with health service users and health governing committee members were conducted. Collected data was analysed through qualitative content analysis.

**Results:**

Study informants reported positive experiences with Payment for Performance and highlighted its potential in improving the availability, accessibility, acceptability and quality of care (AAAQ). However, the study found that persistent barriers for achieving AAAQ still exist in the health system of Tanzania and these contribute to negative experiences of care in the context of P4P.

**Conclusion:**

Our findings suggest that there are a number of positive aspects of care that can be improved by Payment for Performance. However its targeted nature on specific services means that these improvements cannot be generalized at health facility level. Additionally, health workers can go as far as they can in improving health services but some factors that act as barriers as demonstrated in this study are out of their control even in the context of Payment for Performance. In this regard there is need to exercise caution when implementing such initiatives, despite seemingly positive targeted outcomes.

## Introduction

Tanzania is one of many low income countries committed to universal health coverage (UHC) [1] and Sustainable Development Goals (SDGs) [2]. Despite these bold goals, the country was off track in meeting Millennium Development Goals (MDGs) 4 and 5, on child and maternal health [3]. Progress was particularly slow in MDG 5 [4]. This lack of progress prompted the Government of Tanzania to look for ways and strategies to improve health outcomes in these goals. Prompted by a widespread perception among leading policy makers that Payment for Performance (P4P), which promotes the use of financial incentives to improve health worker motivation, is the panacea for the unmet needs in the health sector of many low income countries [5], Tanzania tried this increasingly popular path. P4P is defined as the “transfer of money or material goods conditional on taking a measurable action or achieving a predetermined performance target” [6], and is one type of financing initiative in the basket of Results-Based- Financing (RBF) [7, 8] . Health systems of low income countries, including Tanzania, face huge challenges that limit the access, utilization and quality of health care. While P4P primarily targets the motivation of health workers, it is argued that P4P schemes can act as a catalyst of change in strengthening the health systems of low income-countries [5]. This overarching promise within P4P schemes makes it appealing to politicians and policy makers alike.

Studies conducted in low income setting are inconclusive on the effectiveness of P4P schemes. Some studies have found positive effects of P4P [9, 10], especially in indicators that were strongly under the control of health personnel [9]. While these studies are among those pointing to the potential of P4P in improving the utilization of health services, evidence that P4P works in health systems in low-income to middle-income countries (LMIC) is still highly uncertain and scholars in the field call for caution [6, 11]. Some studies even indicate that P4P schemes might have undesirable effects in health systems with weak infrastructure and leadership and may promote unintended behaviors, create distortions, promote gaming and encourage dependency on financial incentives [6, 12–14]. Furthermore, the costs of running a P4P scheme in low-income settings has been reported to be substantial, at least in the short term, which again fuel debate on the sustainability of such schemes [15]. To this end, given the limited evidence on P4P, one study asked a poignant question regarding these initiatives: is it time to rethink the use of RBF [16] in LMICs?

Tanzania carried out a P4P pilot in the Pwani region [17]. A number of studies have been published as part of a process, impact and cost-effective evaluation of the pilot [15, 18–20]. Less attention has been paid to experiences related to the utilization of health care in this context of P4P which, we argue, is equally important for policy agenda setting and for LMICs thinking or in the process of potentially scaling up these initiatives. Hence, in this study, we aim to explore experiences related to the availability, accessibility, acceptability and quality of care in the context of P4P in Rufiji district of Tanzania from the perspectives of health workers, service users and community health governing committee members.

### P4P in Tanzania

The national health policy in Tanzania emphasizes equity in access to health care [21] and birth care services should be offered free of charge at all public health facilities at all levels [22]. The country has a well-established network of health facilities and referral systems compared to many countries in the sub-Saharan region. Estimates indicate that about 90% of the population in Tanzania lives within 10 km of a health facility, but the quality of the services is generally low at the primary care level. There is an urban bias in the distribution of higher level health facilities disadvantaging the rural population in accessing health care in general and women in accessing emergency obstetric care in particular [23].

Despite widespread access to health facilities, Tanzania has not been able to meet some of its health-related global goals. The country has a high maternal mortality ratio of 410 per 100, 000 live births in 2015, down from 529 per 100,000 live births in 1996 [4, 24, 25]. In fact, Tanzania along with 10 other countries, five of which are in sub-Saharan Africa, were in 2008 responsible for 65% of all maternal deaths in the world [26].

In order to improve health outcomes in maternal and child health and achieve progress towards Sustainable Development Goal number 3 (as targets 3.1 and 3.2), which focus on good health and well-being [2],the Government of Tanzania implemented a P4P pilot in the Pwani region [17, 20]. The pilot was funded by Norway and managed by Clinton Health Access Initiative (CHAI) [17]. Indicators for the Payment for performance pilot were drawn from reproductive and child health (RCH). The indicators covered the following areas: antenatal care, institutional deliveries, post-natal care, prevention of mother-to-child transmission of HIV (PMTCT), family planning and health management information systems (HMIS) [17]. Appendix 1 gives an overview of the P4P indicators. The indicators chosen were aimed to making RCH services available, accessible, acceptable and of good quality, therefore it is important to learn from the experiences of health workers and services users regarding these elements of care.

### Theoretical framework

#### The AAAQ framework

States are obligated under international treaties to safeguard their population’s right to health. The Convention on the Elimination of All Forms of Discrimination Against Women (CEDAW) and the International Convention on Economic, Social and Cultural Rights (ICESCR) are among the treaties that guarantees the right to health. Under these treaties, States have three major obligations: to respect the right to health, to protect their citizens from any infringement to the right to health and to fulfil the right to health [27, 28]. States are obligated to take positive steps to realize this right to health, and such steps may include policy, legislative, budgetary and administrative [27].

Health services are to be *available, accessible, acceptable and of adequate quality* (AAAQ framework) [27, 29]. In order to achieve the right to health, AAAQ must be realized at all levels of care. The first A in the framework, calls for the *availability* of adequate number of goods, services and facilities necessary to provide health care, as well as sufficient number of qualified personnel to staff the services. The second A calls for health services to be *accessible*, both in physical and economic terms. The third A, *acceptability*, calls for health services to be to be respectful of culture of individuals, minorities, peoples and communities. Lastly, the Q in the framework is concerned about health services being medically appropriate and of good *quality* [27, 29].

The AAAQ framework is useful in monitoring health services in general and maternal and child health services in particular [27]. When a health system does not meet the state of AAAQ in maternal and child health services, it contributes to a number of delays in accessing timely care. In Tanzania, there is an explicit connection that poor maternal health services lead to delays in care seeking or accessing such care [30, 31] and as a result the design document for P4P uses the 3-delays model [32] in maternal care as a theoretical basis for P4P. The 3-delays model identifies barriers to access timely maternal services. The delays include 1) decision making to seek care, 2) the identification and getting to the health facility and 3) delays in obtaining adequate quality care upon arrival at the facility [32]. These different phases of delays are very much inter-linked, for example, a poor experience at a health facility (3rd delay) can influence someone’s decision on whether or not to use facility delivery (1st delay).

The government of Tanzania selected P4P indicators from areas critical that can improve AAAQ and potentially reduce the 3-delays in maternal and child care [17]. The areas are: family planning, antenatal care, childbirth/deliveries and basic or comprehensive emergency obstetric care [17] (cf. Appendix 1). Therefore, P4P in Tanzania has to be understood as part of a wider effort by the government to improve maternal and child health services to meet the state of AAAQ. In this study we conducted discussions with health services users, health workers and health governing committees, to examine if P4P was meeting this intended objective.

## Methods

### Study context

The study was carried out in Rufiji, one of the seven districts in the Pwani region. Rufiji is a rural district and according to the 2012 national census, the district had a population of 217,274 [33]. Administratively, Rufiji is divided into 26 wards [33]. The main economic activity of the district is agriculture and 78% of the inhabitants actively participate in this sector. The main cash crops in the district are cashew nuts, coconut and simsim [34]. The district has a total number of 64 health facilities, including two hospitals, five health centres, and 57 dispensaries [34]. Like many rural districts in Tanzania, Rufiji district faces significant shortages of staff and of the 583 positions in the district health sector, only 301 are filled, a total shortage of approximately 49% [34]. Shortages of qualified staff is more pronounced at low level health facilities, i.e. dispensaries and health centres [35] and among all cadres except the medical attendant category which is overrepresented. Rufiji has a delta zone and during rainy season access to health facilities in the delta is difficult and these health facilities periodically face huge problems in procuring medical supplies and in maintaining regular communication with the district health offices located at the district centre Utete.

### Data collection and analysis

A qualitative study approach was adopted using focus group discussions (FGDs) and in-depth interviews (IDIs). Data was collected by the first author with the help of a research assistant for a total of six months period, between January 2013–June 2016). Data was collected at 11 health facilities, including two hospitals, two health centres and seven dispensaries. Of these 11 health facilities, four were church-run and seven were public.

#### FGD with community members

As the study aimed to elicit the experiences and perceptions of health service users on access and quality of care, it was considered important to conduct FGDs with community members. Under this category of participants, we had discussions with mothers and health facility governing committee (HFGC) members. A total of nine FGDs with 44 participants were conducted, seven with mothers and two with HFGC members, for an overview, see Table 1. All focus groups had an average of five participants, a number encouraged in the literature to increase the range of participation of each participant [36]. FDGs with HFGC members had least participants as these committees have up to five members in total. Mothers were targeted primarily for their in-depth experiences with maternal and child health services. In order to maximize the relevance and quality of our data from this group, we used two criteria to recruit them. Firstly, we targeted women who had at least two children with the older one being at least 5 years old. Secondly, we recruited women who had been residing in the same area (catchment area of a particular health facility) for at least 5 years. The participants were recruited with the help of health workers at health facilities. In practice, we ended up including some informants who could not meet one of the criteria; however the majority of our participants were able to meet the criteria.

**Table 1:** Overview of FGDs with health service users

FGD number	Category	No of Participants	Average number of children	Average age
FGD 1	Mothers	6	5	34
FGD 2	Mothers	5	4	31
FGD 3	Mothers	6	5	30
FGD 4	Mothers	6	3	30
FGD 5	Mothers	4	4	31
FGD 6	Mothers	5	3	23
FGD 7	Mothers	5	5	31
FGD 8	HFGC	4	n/a	n/a
FGD 9	HFGC	3	n/a	n/a

The second group of FGD participants was the health facility governing committee members. These committees represent community interests at health facilities. The committees were established in Tanzania in 1999, alongside Community Health Fund (CHF). When P4P pilot was introduced, the committees’ duties were expanded to include monitoring if P4P was implemented in ways protecting the interests of the community. The committees consisted of up to 5 members and many of them were very active in community activities and some even hold local political positions. In addition, the members are knowledgeable about the health status profile of their villages and the district. Typically, the person in charge of a health facility acts as the secretary of the committee. To avoid conflicts of interest the secretary was not invited into the FGD with the committee members. In FDGs, a topic guide was used with great flexibility to allow the discussion of emergent issues. The topic guide covered the following issue: experiences with MCH services, quality of health care, access and utilization of health services.

#### IDI’s with health workers

To complement the data from the FGDs with community members, we conducted 31 IDIs with health workers of different cadres ranging from medical officers, assistant medical officers, clinical officers, nursing staff, laboratory staff and medical attendants were conducted. Table 2 provides an overview of study informants.

**Table 2:** Overview of IDIs

Category of informant	Number of interviews
Medical Officers (MO)	2
Assistant Medical Officers (AMO)	2
Clinical Officers (CO)	3
Nursing staff	11
Medical attendants (MA)	11
Laboratory staff	2

All FGDs and interviews except two interviews with Medical Officers were conducted in Swahili. The first author speaks colloquial Swahili, while the research assistant is a Tanzanian citizen with experience in qualitative health services research. All interviews and FGDs were recorded, transcribed in Swahili and then translated into English. In addition, rapid note taking was used. Translations were error checked. Qualitative content analysis was used as the mode of analysis [37], Table 3, gives an overview of the analysis process. The transcripts were subjected to a thorough review before the coding exercise began. Meaning units at manifest level were identified and coded and from these sub-themes and themes were identified. OpenCode 3.6 software [38] was used for data management.

**Table 3:** Examples of moving from meaning units to themes in content analysis of health service users’ FGDs

Meaning unit	Condensed meaning unit Description close to the text	Condensed meaning unit Interpretation of the underlying meaning	Sub-theme	Theme
Nurses here used to use bad languages to us but now they are getting better. They welcome us in a polite language, not like before when they used to shout at us in the labor room. These days if you feel the pain and call them, they come and listen. Before they used to scream at us and treat us as children	Improving staff attitudes	Nurses being more respectful to patients	Experiences with RCH services	Experiences with health care services in the context of P4P
When I gave birth in 2006 there was a tendency were nurses can leave you unattended in labor. They would ask you whether it is your first birth or not and if it wasn’t then they can leave you.	Nurses leaving patients unattended	Nurses abandoning care		
RCH services are good when you come here you are treated in a good way until you give birth and discharged. The out-patient department and other places are still the same; at RCH we can see more tools and medicines coming	RCH services are better	RCH offering better services		
Kwa kweli the relationship among nurses here is good, before we used to leave this place at 4 pm, but now we are getting our services timely. We don’t stay here for long now, its only today we are still here because they had a meeting	Waiting time is becoming less	Shortened patient waiting time		
It’s hard to find money, we are asked to buy the delivery kit and many of us can’t afford this. If you come without this you don’t get help.	Cannot afford care	Lack of money	Barriers for accessing care	
The facilities here are poor; first and foremost, the toilets are very dirty, we don’t even know who is supposed to clean them. If you decide to go there right now, you won’t believe your eyes.	Dirty toilets	Poor facilities		
What I heard is that it is a must for every pregnant woman to come with her own equipment for birth. I do not understand if the government is not providing these things	Pregnant women buying own delivery kit	Lack of equipments & supplies		
We used to have one medical attendant doing everything including what the doctor is doing now. We always wish if the government can help us by providing another staff, maybe we can get help quickly	Few health workers available	Lack of enough staff

**Table 4:** Overview of P4P indicators of the Pwani Pilot, Tanzania

Service Category	Indicator
Family Planning, Healthy Timing and Spacing of Pregnancy	Couple Year Protection Rate (CYP)
Focused Antenatal Care	% of ANC clients receiving IPT2 (Malaria prophylaxis coverage)
PMTCT	% HIV positive ANC clients receiving ARVs for prophylaxis
Labour & Delivery	% of facility based deliveries
Labour & Delivery	% of completely and properly filled partograms
Newborn Care	% of newborns receiving OPV0 in the first two weeks of life
Postpartum Care	% of newly delivered mothers attending postnatal clinic within 7 days after delivery
Child Health	% of Children under one year old receiving Penta Valent 3
Child Health	% of children under one year old receiving measles vaccines
Maternal and Newborn Mortality	% of maternal and newborn deaths that are appropriately audited on time
Health System strengthening	% of facilities reporting stock-outs of either one or more of the tracer medicines in a specified period (< 8 days)
HMIS strengthening	HMIS monthly reports correctly filled and submitted on time to CHMT (by 7th of the following month)
HMIS strengthening	% of facilities included in the HMIS monthly reports exported through District Health Information System (DHIS) to RHMT in timely manner (by 14th of the following month)
HMIS strengthening	% of districts included in the HMIS monthly reports exported through DHIS to MoHSW on time (by 21st of the following month)
Management	Submission to MoHSW of a Semi-Annual Regional Health Profile report, based on DHIS
Management	% of facilities receiving a copy of a Quarterly District Health Profile report, based on DHIS Overall performance along
Overall	P4P facility-based indicators

## Research ethics

The study was registered in Norway through the Norwegian Social Science Data Services (NSD) and in Tanzania through the Ifakara Institutional Review Board (IHI/IRB/No: 24–2012) and the National Institute for Medical Research (NIMR/HQ/R.8a/Vol.IX/1515). Consent was sought after and given and we have depersonalized data by labeling facilities by letters, and informants by titles.

## Results

This section presents major themes that emerged in FGDs and IDIs with health service users and health workers related to their experiences with and perceptions of the availability, accessibility, acceptability and quality of care with health services. On one hand, users noted improvements in health service delivery in terms of the availability, accessibility and acceptability of health services mainly from the RCH department. They reported increased availability of medicines provided free of charge, which also mean they have to spend less money in buying these medicines, which improves the accessibility of these services. In addition, health service users reported positive staff attitudes which were seen as important in the acceptability of health services. On the other hand, numerous long term challenges within the health system of Tanzania were reported to still persist after the introduction of P4P, hindering access to and provision of quality health care. The following section presents these results.

### Perceived improvements of access to and quality of care in the context of P4P

The study participants reported a number of notable improvements to the access and quality of care. Like many countries, Tanzania has been investing in improving health outcomes and perhaps some of these benefits are beginning to trickle down to intended beneficiaries, the health service users. In our study, however, it is important to take note that some of these positive changes may be attributed to the P4P whose main aim is to improve access to and quality of care in targeted service areas. In this section, we provide accounts and citations from our study participants pointing to these perceived improvements.

#### Perceived improvements in staff attitudes

Many studies in Tanzania and sub-Saharan Africa have found negative staff attitudes to be contributory to barriers in accessing health care in general and maternal care in particular [39, 40]. Our study provides a different picture. The study participants reported to have experienced a positive change in staff attitudes in maternal and child health care after the introduction of P4P, while concerns were still raised on poor staff attitudes in other departments not targeted by P4P, see for example our earlier publication on the same context [41]. Particular attention was brought to the use of language as illustrated by the following citation:

*“Nurses here used to use bad languages to us but now they are getting better. They welcome us in a polite language, not like before when they used to shout at us in the labor room. These days if you feel the pain and call them, they come and listen. Before they used to scream at us and treat us as children.”* (Mother 1-FGD 3)

Health workers we interviewed on this issue agreed that such positive change in attitudes were observable and constituted a strategy on the part of the health facility and RCH teams to meet P4P indicators as illustrated in the following extract:

*“The program to a certain extent also changed our attitude towards patients because in the past there were so many complaints from patients but not that much anymore. We encourage each other to use polite language. If we don’t then patients can go somewhere else and then we don’t get P4P money.”* (Nursing staff-IDI)

Changed health worker attitudes were also expressed in their presence during labour and birth. There were numerous reports that previously women were left unattended in the labor room and according to FGD participants this was seen to be changing as one mother noted:

*“Nurses could leave you unattended in labor. They would ask you whether it is your first birth or not and if it wasn’t then they could leave you saying ‘you have labor experience’ and yet every labor experience is different.”* (Mother 2-FGD 4)

A number of studies in sub-Saharan Africa have shown that changes in the behaviors of health workers can happen when incentives are introduced [12, 13] and these changes can be positive or negative to the quality of health care. P4P may encourage competition among health facilities sharing the same catchment area. Health service users can choose to access care at health facilities which offers better quality. While some aspects of quality care are beyond the control of individual health workers, some aspects, like use of polite language and presence (for compassion) are possible to influence. Health workers are very well aware that building trust with health service users is one of the immediate strategies they can use to improve the utilization of health services and thus meeting P4P indicators in targeted service areas.

#### Improved service delivery in RCH

Health service users noted that service delivery had improved in certain services targeted by P4P. This view was also shared by health workers interviewed. The P4P pilot was drawing its indicators from the RCH department. In this regard there were noted differences in the delivery of service in the RCH and non-RCH departments. As one mother noted:

*“RCH services are good. When you come here you are treated in a good way until you give birth and are discharged. The out-patient department and other places are still the same; at RCH we can see more tools and medicines coming.”* (Mother 5-FGD 1)

The P4P bonus given to health facilities was divided into two parts, one part was for individual health workers (75–90%) and another part (10–25%) was used for demand creating activities, for example buying of important medicines in meeting P4P targets. It was this money for demand creating activities that slightly improved the tools or medicines situation as noted by service users. During fieldwork, we were shown medicines purchased with this money or renovations carried out with this money. Health workers attributed the improvements in service delivery to this fund. As one informant noted:

*“The quality of service has improved at the RCH and not here at the out-patient department. For example, at RCH patients are getting medicine on time including delivery kits, and here at out-patient department, they have to buy their own medicine, injections, among other things. So some will say why waste my time going to the hospital? The program is good only that it selects certain areas.”* (Medical Attendant - IDI)

#### Improved teamwork in the RCH

According to both service users and providers perceived improvements in the quality of services were observed only in P4P targeted areas. It was also reported that RCH staff seemed to be working together much better as a team and more efficiently as illustrated in the following extract:

*“Kwa kweli [truly] the relationship among nurses here is good, before we used to leave this place at 4 pm, but now we are getting our services timely. We don’t stay here for long now, its only today we are still here because they had a meeting”.* (Mother 4-FGD 3)

Health workers agreed that P4P had contributed to the new teamwork spirit. P4P in Tanzania pays individual health workers based on team effort, that is, the incentive is based on performance achievement in targeted RCH services. The following citation illustrates this need for cooperation among teams:

*“P4P has made us work closer compared to before where everyone was filling his/her report alone. We work as a team now and this has improved our working relationship because we now divide our work equally. However, the workload has also increased, especially for us in the RCH as we have many indicators to meet the targets for bonus.”* (Nursing staff-IDI)

#### Improved health education

Another area that was reported to have significantly improved within the RCH department was health education. Mothers reported that health workers were using substantial time imparting health knowledge both at health facilities and during outreach programmes as the following citation illustrates:

*“We are happy because we get health education and we are told what to do and when. This knowledge is important because when you find your child in a certain condition you know what to do. This is helping our children to be healthy”.* (Mother 2-FGD 1)

Outreach programmes and health education are among the strategies health workers can use to increase demand in P4P targeted services. However, this may also have spillover benefits to other non-targeted services. For example, health workers reported that they normally carry out outreach programmes for RCH services but in the process impart useful knowledge on malaria prevention.

#### Improved data management

Data management was noted to have improved due to P4P. Health management information systems (HMIS) in many sub-Saharan African countries are poor and this impact on the quality of care to patients. Without the knowledge of a patient’s medical history, it is difficult for health workers to offer proper and adequate personalized care. The P4P in Tanzania is perceived by health workers to improve data management which is important for quality care. As noted by one informant:

*“I have been a member in the last three committees for this facility. For my facility I can see that there are improvements. These can be seen in medical supplies for RCH, general service delivery, mainly because of this 25% we get from P4P. We also see improvements in data management and performance figures, and as you can see we put a chart out for people to see these improvements”.* (HFGC member- FGD 8)

In our interviews with health workers, improved and accurate data management was noted as an important factor for receiving P4P bonuses. As illustrated in the following citation:

*“You see, before P4P the issue of preparing our reports was not taken seriously, we were told the deadline to submit the reports is the 5*^*th*^ *of every month but we were not submitting them in time. But now even before the 1*^*st*^ *of every month we are already starting to prepare and even submit them.”* (Clinical Officer-IDI)

While the primary motive for health workers to timely submit reports is driven by the need to get P4P bonuses, arguably correct data management goes a long way in improving the HMIS and better inform policy makers on policy options to follow.

### Persistent barriers to the access to and quality of care in the context of P4P

Despite some noticeable improvements and positive experiences with regards to the access to and perceived quality of RCH services in the majority of health facilities we visited, many barriers to the utilization and access of health care were reported by health service users and health workers. It must be emphasised that these persistent barriers are usual structural health system aspects but nevertheless discussed by research participants in the context of P4P. Provider side factors were reported as main barriers. However, study informants also noted some user side factors as barriers to the access of RCH services. This section predominantly present these provider side barriers for the access of quality care which vary from resource availability, infrastructure and remuneration. On the user side factors, two main barriers were mentioned, which are, lack of money and long distances to facilities.

#### Poor infrastructure

A number of health facilities were not in good condition, and did not have adequate infrastructure to support adequate service provision. Lack of privacy and hygienic conditions were the main concerns for health workers.

*“I will take you to the labor room for you to see the environment we work in, there is no privacy here, and everyone hears and sees everything. Even the toilets are broken. Our counselling and testing centre is there at the doctor’s office and we use our out-patient department as the storeroom.”* (Nursing staff-IDI)

Unsatisfactory infrastructure was also noted by health service users in our focus group discussions. These poor facility conditions impacted on the utilization of health services:

*“The facilities here are poor; first and foremost, the toilets are very dirty, we don’t even know who is supposed to clean them. If you decide to go there right now, you won’t believe your eyes. Now the problem is that you may stay here but by the time you are discharged you can carry another disease from this place, and please don’t even try to drink water from here, it’s dirty. Is this place supposed to make people feel better or sick?”* (Mother 4-FGD 6)

Lack of clean water was also noted as a main challenge for a number of facilities in Rufiji district, and in some cases, patients have to carry clean water to health facilities for use when taking pills. Lack of clean water at health facilities in Rufiji compounds the problem in a district where water- borne diseases are a challenge especially in the delta areas.

#### Shortages in drugs and medical supplies

As a typical rural district in Tanzania, Rufiji suffers from shortages in drugs and medical supplies. Although improvement has been noted in drugs supplies related to P4P targeted service areas, the general situation of drug and medical supplies remains unsatisfactory both to the health workers and health service users. As noted in the following extract:

*“Working equipment is a big problem. Sometimes we even lack basic things such as gloves in the labor room. You know infections are a serious problem at hospitals, but sometimes we don’t even have detergents to clean the equipment we have. In addition, there are constant drug stock-outs, normally we are supposed to receive medicines every quarter but sometimes we can even reach half a year with no medicines”* (Clinical Officer-IDI)

Shortages of medicines and drug stock-out are a constant problem in the health system of Tanzania. In our focus group discussions with mothers, this was noted as a main barrier for health facility delivery:

*“For my first birth I did not deliver here but what I heard is that it is a must for every pregnant woman to come with her own equipment for birth. I do not understand if the government is not providing these things or if the nurses are selling the supplies to pharmacies, because it doesn’t matter if it is in the beginning or end of the month. Everyone buys something to deliver here and in addition we pay some cash after delivery, and this doesn’t matter whether it is a government of private hospital, so what do you do when you don’t have money?”* (Mother 1-FGD 7)

The unavailability of essential medication and equipment at health facilities affect the relationship between health workers and health service users. Birth services are principally free in Tanzania, but the policy discourse differs from realities at health facilities and in many cases, health service users’ suspects that health workers misuse or steal drugs and essential equipment as the above citation from an FGD seem to indicate.

#### Lack of qualified and adequate staff

Human resources for health is a major challenge in Tanzania, likewise, health facilities in Rufiji face this huge challenge of staff shortages, with the majority of health facilities having less than 50% workforce. The result is a lot of work load for the few health workers available:

*“We are only two workers here, but we serve almost 8 000 people from more than three villages. You can see that our workload is so huge, we try to help each other with everything but still it is too much work. Maybe now there is this P4P, but besides that there is little to keep us motivated in this job.”* (Clinical Officer-IDI)

In our FGDs with health service users, understaffing and lack of adequate skills was noted as a huge barrier to get timely and appropriate services:

*“I woke up feeling sick today and then I decided to come to the dispensary early enough for help, but as you can see I am still here, I haven’t seen the doctor yet. But maybe it’s even better these days, before we used to have one medical attendant doing everything including what the doctor is doing now. We always wish if the government can help us by providing another staff, maybe we can get help quickly”* (Mother 3-FGD 2)

While as noted earlier barriers to the utilization and access of quality care were identified from the provider side, as most of questions in interview guides were mainly exploring RCH services in the context of P4P, a provider side initiative in the Tanzanian context. Regardless, a few user side factors were also mentioned as barriers to the access of RCH services and these were lack of money and long distances to health facilities.

#### Lack of money and long distances

Lack of money was mentioned as a main barrier for the utilization of health care in general and maternal health care in particular. Although maternal care is principally free in Tanzania women pay or contribute significantly to the costs associated with birthing services. As reported by one mother:

*“It’s hard to find money, we are asked to buy the delivery kit and many of us can’t afford this. If you come without this you don’t get help. You will find that you may need to have between TSH 15 000- 20 000, this is a lot of money, where do you get it?”* (Mother 6 – FGD 4)

While it was noted that there were some improvements in the supply of medicines specifically related to P4P indicators, this did not improve the overall shortages and as such service users still had to in certain circumstances buy equipments and medicines that should be provided for. Due to these costs, many women end up delivering at home with the assistance of traditional birth attendants (TBAs). In addition to cost, distance played a major role in limiting access as is illustrates in the following quote:

*“Some of us live far from here, sometimes when our delivery time is near we come to live with relatives near a health facility, but not all of us have these relatives. Reaching here is a problem because we don’t have good roads and transport. Imagine using a bicycle as a means of transport when you are in labor. There are some small dispensaries around but some of these are of no help.”* (Mother 2- FGD 5)

Given this environment, P4P was introduced in a context of many systemic challenges, maybe with the hope that some of these challenges will be alleviated. To health workers, the challenges present some of the reasons why P4P targets are not met whereas for health service users the challenges presents barriers for accessing and utilizing health services.

## Discussion

Our findings are dominated by two main themes, which are, perceived improvements to the access of and quality of care in services targeted by P4P and persistent barriers to the utilization, access to and quality of care to health services targeted and non-targeted by P4P. The first two sections of our discussion will shed light into these two themes by looking at the state of AAAQ in the context of P4P. In the third section of our discussion, we will discuss the possible implications of P4P to access to, utilization of, and quality of care.

### Improved access and service delivery

AAAQ framework stresses the need for adequate health infrastructure and services to be available within a geographical area, accessible physically and economically, acceptable culturally and ethically, and be of good quality [27, 29]. Our study suggests that P4P can improve some aspects of AAAQ, which will be highlighted in this section.

Through the accounts of mothers and health workers, our study reported improvements in staff attitudes towards RCH patients. Poor staff attitudes have been reported in many studies in Tanzania and sub-Saharan Africa [39, 40, 42, 43] as one of the barriers for facility delivery. Negative experiences at health facility influences the decision on whether women seek professional care or not [30, 31] thereby contributing to the first two delays in the 3- delay model [32].

In addition to improved staff attitudes, our study reported improvements in service delivery in services targeted by P4P. These improvements were noted by both health services users and health workers. Health workers attributed these improvements to the facility fund (approx. 25% of the bonus money health facilities receive from P4P). The fund has to an extent help to curtail shortages of essential drugs and medicines, and had contributed to the improved infrastructure in the RCH department. Studies in Tanzania had reported shortages in medical supplies, essential drugs and poor facilities in the MCH as barriers to the utilization, access to and quality of care [30, 31, 39] as these contributed to women’s poor experiences at health facilities. In this regard, slight improvements in the quality of care were welcomed by health service users.

Reported improvements in staff attitudes and service delivery significantly improves the trust between health providers and users as lack of it in the health care system of Tanzania has been reported as a major barrier to the utilization of health care [39]. Trust in the health care system, may improve the 1st, 2nd and 3RD delays in maternal care, as aimed by the P4P pilot [17, 32]. However, these positive aspects, arguably, are not overarching and may not shift the state of AAAQ in Tanzania significantly, as discussed in the following section.

### Persistent barriers to the access to and quality of care

Persistent barriers to the access to and quality of care were reported in our study and the majority of these are health systems challenges. The main challenges that were identified from the user side are: lack of money, lack of transport and/or long distance to the health facility. These challenges had been reported in previous studies in Tanzania as impediments to seek care [39, 40, 42]. The P4P pilot in Tanzania targets the provider side with the assumption that it is health systems challenges that are mainly contributory to the lack of utilization of health care services. To an extent this seems to be corroborated by our study findings. However, given that there is a huge discrepancy between ANC figures in Tanzania, which stands at 96% and facility deliveries, which stands at 50% [24], there could be a reason to suggest that there are user side factors, such as community preferences which need to be targeted if the country needs to see improvements on facility deliveries. Community preferences on place of delivery were reported in Tanzania by health workers as something that could make P4P targets less achievable [18]. However, facility delivery seems to be an indicator where P4P has the greatest impact in Sub Saharan Africa [9, 10], suggesting that if this can be sustained facility delivery can significantly improve in this context, including Tanzania.

While community preferences may play a role, health systems factors remain as the main barriers for the access to and utilization of care. Resource availability including inadequate infrastructure, pack of qualified and adequate staff, and lack of medicines and adequate or functioning equipment were reported by health users and providers in our study as main barriers to the access of care. Health workers identified these as contributory for not meeting P4P targets, and this finding in corroborated in another study in Tanzania [18].

### Payment for performance: implications to access, utilization and quality of care

Our study findings suggest that provider P4P can improve certain aspects of care. In the sub-Saharan African context, three evaluations in Rwanda, Burundi and Tanzania concluded that P4P can improve some aspects of health service delivery [9, 10, 44], the findings of these studies suggest that P4P has a strong effect on services where health personnel had a direct control on indicators, for example, indicator on facility deliveries. Our study has also demonstrated that health workers were able to change their behavior by improving their attitudes and relations in general with patients at the RCH department targeted by P4P. The use of positive strategies, such as positive attitudes and health education were reported in Tanzania [42]. What is important to note is that P4P or other forms of incentives have the power to alter the behavior of health workers. Positive strategies goes a long way in improving AAAQ, however, it could be useful to reflect on the possible pitfalls that may result from this alteration of behavior, which can equally be detrimental to AAAQ.

P4P can have unintended consequences which negatively affects the quality of care. One study in Tanzania found out that health workers resorted to coercive strategies for women to deliver at health facilities [12]. Another negative consequence P4P may have on AAAQ is when it leads to ‘gaming’, that is, the neglect of non-remunerated aspects of care work [45]. Narratives from users and providers showed that services in the RCH department are prioritized than non-targeted services. Hence improvements in service delivery were only noted in the RCH department. The prioritization of health care services can have a negative effect on the state of AAAQ. A number of factors contribute to this in our study context. Firstly, P4P pilot design in Tanzania is focused on RCH services only, which provides a platform for prioritization of these services, secondly, the incentive distribution modality favors workers working with RCH services, which as reported in one study in Tanzania, affects teamwork and social relations at health facilities [41].

It has been argued that P4P need to have a health system thinking than being too targeted on a few services and indicators [46] and equally there have been recent calls to reconsider the use of Payment for Performance in the health sectors of LMICs [16]. Although this paper reported some positive aspects of P4P, it seems the persistent structural health system challenges and unintended consequences caused by P4P [12, 41, 47] outweigh the benefits.

## Limitations

This study aimed to explore wider experiences with P4P, hence to a certain degree themes presented under positive experiences and wider systemic barriers could overlap and potentially conflictual. It must be stressed that the positive aspects were derived in particular sections, RCH, implementing P4P and not representative of wider health facility experiences. The study was conducted in only one district out of six in the Pwani Region that implemented the P4P pilot, however, the general characteristics and health status profile of Rufiji district does not differ with the rest of the region, except for the health facilities in the delta zone, which were not included in our sample due to accessibility challenges. In addition, the study informants and participants were purposeful selected, at times with the help of health facility staff, this implies that their viewpoints may not be representative of other individuals or settings. Lastly, FGDs with health committee members had few participants mainly because these committees have up to 5 members. In our case at the two facilities we visited, these committees had less than 5 members.

## Conclusion

In conclusion, our study showed that P4P can lead to the improvement of health service delivery. These positive aspects of P4P can be harnessed and integrated in the health systems of low-income countries. However, particular attention needs to be paid to potential unintended consequences of P4P. In situations where health workers have limited resources to reach performance targets, P4P may encourage behaviors that not consistent with the need to achieve AAAQ in health service.

## Data Availability

Not applicable.
